# Genetic predisposition to male breast cancer in Poland

**DOI:** 10.1186/s12885-021-08718-3

**Published:** 2021-08-30

**Authors:** Marek Szwiec, Joanna Tomiczek-Szwiec, Wojciech Kluźniak, Dominika Wokołorczyk, Karolina Osowiecka, Robert Sibilski, Małgorzata Wachowiak, Jacek Gronwald, Helena Gronwald, Jan Lubiński, Cezary Cybulski, Steven A. Narod, Tomasz Huzarski

**Affiliations:** 1 Department of Surgery and Oncology, University of Zielona Góra, Zyty 28, 65-046 Zielona Góra, Poland; 2 Department of Histology, University of Opole, Oleska 48, 45-052 Opole, Poland; 3 Department of Genetics and Pathology, International Hereditary Cancer Center, Pomeranian Medical University, 71-252 Szczecin, Poland; 4 Department of Psychology and Sociology of Health and Public Health, School of Public Health, University of Warmia and Mazury in Olsztyn, Al. Warszawska 30, 11-041 Olsztyn, Poland; 5 Department of Clinical Oncology, University Hospital in Zielona Góra, Zyty 26, 65-046 Zielona Góra, Poland; 6 Department of Propaedeutics, Physical Diagnostics and Dental Physiotherapy, Pomeranian Medical University in Szczecin, Poland, al. Powstańców Wielkopolskich 72, 70-111 Szczecin, Poland; 7 Women’s College Research Institute, Women’s College Hospital, University of Toronto, Toronto, ON M5G 1N8 Canada; 8 Dalla Lana School of Public Health, University of Toronto, Toronto, ON M5T 3M7 Canada; 9 Department of Clinical Genetics and Pathology, University of Zielona Góra, Zyty 28, 65-046 Zielona Góra, Poland

**Keywords:** *BRCA1*, *BRCA2*, *PALB2*, *CHEK2*, *NBN*, *RECQL*, Mutation, Male breast cancer

## Abstract

**Background:**

Breast cancer in men accounts for fewer than 1 % of all breast cancer cases diagnosed in men and women. Genes which predispose to male breast cancer include *BRCA1* and *BRCA2.* The role of other genes is less clear. In Poland, 20 founder mutations in *BRCA1*, *BRCA2*, *CHEK2*, *PALB2*, *NBN*, *RECQL* are responsible for the majority of hereditary breast cancer cases in women, but the utility this genes panel has not been tested in men.

**Methods:**

We estimated the prevalence of 20 alleles in six genes (*BRCA1*, *BRCA2*, *CHEK2*, *PALB2*, *NBN*, *RECQL)* in 165 Polish male breast cancer patients. We compared the frequency of selected variants in male breast cancer cases and controls.

**Results:**

One of the 20 mutations was seen in 22 of 165 cases (13.3%). Only one *BRCA1* mutation and two *BRCA2* mutations were found. We observed statistically significant associations for *PALB2* and *CHEK2* truncating mutations. A *PALB2* mutation was detected in four cases (OR = 11.66; *p* < 0.001). A *CHEK2* truncating mutation was detected in five cases (OR = 2.93;*p* = 0.02).

**Conclusion:**

In conclusion, we recommend that a molecular test for *BRCA1, BRCA2, PALB2* and *CHEK2* recurrent mutations should be offered to male breast cancer patients in Poland.

**Supplementary Information:**

The online version contains supplementary material available at 10.1186/s12885-021-08718-3.

## Background

Breast cancer in men accounts for fewer than 1 % of all breast cancer cases diagnosed in men and women [1]. The risk of breast cancer is elevated in men with a *BRCA1* or *BRCA2* mutation [2–4]. The prevalence of *BRCA2* mutations in men with breast cancer ranges from 4 to 40% in different populations [5–13]. The prevalence of *BRCA1* mutations in men with breast cancer is lower; estimates range from 0 to 7% [8, 14–18]. There are reports of male patients with mutations in *PALB2* and *CHEK2* [12, 16, 19–22]. The risk of male breast cancer in carriers of *PALB2* mutations is increased by approximately 7- fold [12, 16, 19, 21, 23–26]. The common truncating allele of *CHEK2* (1100delC) was associated with a 4.5-fold increased risk of the male breast cancer in the Scandinavian population [22].

Poland is a genetically homogeneous population. Twenty founder alleles in *BRCA1*, *BRCA2, CHEK2*, *NBN*, *PALB2* and *RECQL* genes are responsible for the majority of hereditary breast cancers in Polish women. We identified one of these 20 mutations in about half of women with familial breast cancer (the mean number of breast cancers per family was 3.6) [27]. This panel of 20 founder mutations in *BRCA1*, *BRCA2 CHEK2*, *NBN*, *PALB2* and *RECQL* genes included about 85% of all mutations detected in high-risk families in Poland [27]. One of these founder mutations is present in 12.5% in Polish women with breast cancer diagnosed before age 51, and in 17% in Polish women diagnosed at age 40 or below [28–33].

Founder mutations in the *BRCA*, *CHEK2* and *PALB2* genes have been described in the literature in other ethnic populations. Founder *BRCA* mutations have been detected in the population of Ashkenazi women, in Russia, Latvia, Slovenia, Slovakia, Germany, Finland, the Czech Republic, Tunisia, Hungary and the Bahamas, and accounted for 20 to 90% of all detected mutations in these genes [34–45].

The *CHEK2* 1100delC mutation is present in Eastern and Northern European populations with a frequency of 0.2–1.4% in the general population [46, 47]. Two other truncating mutations of *CHEK2* (c.444 + 1G > A, del5395(ex10-11del)) are predominantly detected in Eastern European countries, (c.444 + 1G > A in Poland, Belarus, Russia; and del5395(ex10-11del) in Poland, Belarus, Czech Republic, Slovakia) [35, 48]. Both alleles are also present at lower frequencies in Germany (0.1% del5395(ex10-11del) and 0.3% for c.444 + 1G > A) [49]. Recurrent *PALB2* mutations have been detected in Finland, Argentina, Spain, China and Canada with a frequency of 0.6–3.6% [50–55].

In the current study, we evaluated the frequency of 20 recurrent mutations in six genes (*BRCA1*, *BRCA2*, *CHEK2*, *PALB2*, *NBN* and *RECQL)* in 165 men diagnosed with breast cancer in Poland.

## Methods

### Patients

Patients were selected from a registry of breast cancer cases held at the Hereditary Cancer Center in Szczecin. We identified 186 men with breast cancer from a registry of 25,000 breast cancer cases diagnosed in Poland. Patients had a genetic consultation between 2000 and 2019 at the Hereditary Cancer Center in Szczecin. During the patient interview a written, informed consent and a blood sample were obtained from all subjects. All patients were of European ancestry and ethnic Poles.

Because of the quality of the DNA we were able to include 165 men in the study (88.7%). Clinical data including family history (cancer cases in first and second-degree relatives) and tumor characteristics were obtained during an interview with the patient and from medical records (Table 1). All clinical data were obtained in more than 70% of cases.

**Table 1:** Clinical characteristics of breast cancers in mutation carriers and non-carriers

Mutation	BRCA1			BRCA2			PALB2			CHEK2 truncating			CHEK2 c.470C > T			NBN			Non-carriers		
	N	Tot	%	N	Tot	%	N	Tot	%	N	Tot	%	N	Tot	%	N	Tot	%	N	Tot	%
Histology																					
Ductal	1	1	100.0	2	2	100.0	3	3	100.0	4	4	100.0	7	8	87.5	1	1	100.0	111	125	88.8
Lobular	0	1	0.0	0	2	0.0	0	3	0.0	0	4	0.0	0	8	0.0	0	1	0.0	6	125	4.8
Other	0	1	0.0	0	2	0.0	0	3	0.0	0	4	0.0	1	8	12.5	0	1	0.0	8	125	6.4
Tumour size																					
T1/T2	0	1	0.0	2	2	100.0	2	3	66.7	3	3	100.0	7	7	100.0	1	1	100.0	83	110	75.5
T3/T4	1	1	100.0	0	2	0.0	1	3	33.3	0	3	0.0	0	7	0.0	0	1	0.0	27	110	24.5
ER positive	1	1	100.0	1	1	100.0	3	3	100.0	4	4	100.0	7	7	100.0	1	1	100.0	100	111	90.0
PR positive	1	1	100.0	1	1	100.0	3	3	100.0	4	4	100.0	7	7	100.0	1	1	100.0	88	110	80.0
HER2 positive	0	1	0.0	0	1	0.0	0	3	0.0	0	4	0.0	1	7	14.3	1	1	100.0	11	108	10.2
Triple negative	0	1	0.0	0	1	0.0	0	3	0.0	0	4	0.0	0	7	0.0	0	1	0.0	11	107	10.3
Lymph nodes positive	0	1	0.0	2	2	100.0	1	3	33.3	1	3	33.3	1	7	14.3	0	1	0.0	53	104	51.0
Bilateral	0	1	0.0	0	2	0.0	2	4	50.0	0	5	0.0	0	9	0.0	0	1	0.0	0	143	0.0
Multiple primary cancers	0	1	0.0	2	2	100.0	2	4	50.0	1	5	20.0	3	9	33.3	0	1	0.0	12	143	8.4
Family history positive																					
Breast	1	1	100.0	1	2	50.0	3	4	75.0	2	5	40..0	2	9	22.2	0	1	0.0	38	143	26.6
Ovarian	0	1	0.0	0	2	0.0	1	4	25.0	0	5	0.0	0	9	0.0	0	1	0.0	6	143	4.2
Other	0	1	0.0	1	2	50.0	0	4	0.0	3	5	60.0	5	9	55.6	0	1	0.0	51	143	35.7
Age of diagnosis																					
< 50	0	1	0.0	0	2	0.0	2	4	50.0	1	5	20.0	2	9	22.2	0	1	0.0	20	143	14.0
≥ 50	1	1	100.0	2	2	100.0	2	4	50.0	4	5	80.0	7	9	77.8	1	1	100.0	123	143	86.0
Median age of diagnosis (range)	74			69 (62–76)			57 (42–77)			62 (33–70)			64 (34–72)			62			63 (26–89)		

### Controls

The frequencies of *BRCA1, CHEK2, PALB2, NBN* in cancer-free controls were taken from previous studies [29, 30, 56]. The frequency of mutations in the *BRCA1* and *NBN* genes were determined in the group 4000 samples described by Lener et al. [56]. The frequency of mutations in the *CHEK2* gene were determined in the group of 5496 samples described by Cybulski et al. [29]. The frequency of mutations in the *PALB2* gene were determined in the group of 4702 samples described by Cybulski et al. [30].

### Genotyping

DNA was isolated from 5 to 10 mL of peripheral blood. Recurrent mutations of *BRCA1*, *BRCA2*, *CHEK2*, *NBN*, *PALB2* and *RECQL* were genotyped as described previously [28–32]. The *BRCA1* 5382insC (c.5263_5264insC) and 4153delA (c.4035delA) mutations were detected using allele-specific oligonucleotide polymerase chain reaction (PCR) and C61G (c.181 T > G) was detected using restriction fragment length polymorphism PCR. The other mutations of *BRCA1*: 3819del5 (c.3770_3704delGTAAA),185delAG (c.68_69delAG), 5370C > T (c.5251C > T), 3875del4 (c.3756_3759delGTCT) and *BRCA2* (c.658_659delGT, c.3847_3848delGT, c.5239_5240incT, c.5946delT, c.7913_7917del5) were genotyped using TaqMan assays (Applied Biosystems/Life Technologies, Carlsbad, CA) on Roche LightCycler 480. The c.444 + 1G > A and c.470C > T in *CHEK2* were detected using restriction fragment length polymorphism PCR. The *CHEK2* del5395(ex10-11del) was tested by a multiplex PCR reaction and c.1100delC was detected by allele-specific oligonucleotide PCR. One mutation on *NBN* was detected using allele-specific oligonucleotide PCR. The two mutations of *PALB2* c.509_510delGA and c.172_175deITTGT and one c.1667_1667 + 3delAGTA of *RECQL* were analyzed using TaqMan assays (Applied Biosystems/Life Technologies, Carlsbad, CA) on Roche LightCycler 480. All mutations were confirmed by Sanger direct sequencing. Sequencing reactions were performed using a BigDye Terminator v3.1 Cycle Sequencing Kit (Life Technologies) according to the manufacturer´s protocol. Sequencing products were analyzed on the ABI Prism 3100 Genetic Analyzer (Life Technologies). The DNA quality allowed for analysis of all 20 alleles in 165 from 186 patients (88.7%). Statistical analysis comparing the frequency of mutations in the cases and controls was performed in this group of 165 cases.

### Statistical analysis

We estimated the odds ratios for selected recurrent mutations. We compared the prevalence of the ten different mutant alleles in *BRCA1*, *CHEK2*, *PALB2, NBN* in the study group and in control groups. Odds ratios (OR) were generated from two-by-two tables and statistical significance was assessed using Fisher exact test or Chi-squared test with Yates correction. ORs were used as estimates of relative risk.

## Results

There were 165 men with breast cancer included in the study. The median age of diagnosis was 62 years (range 26–89 years). Twenty-five cases were diagnosed before age 50 (15.2%) (Table 1). The majority of cases were diagnosed with ductal carcinoma (89.6% of cases), followed by lobular (4.2% of cases) and papillary cancer (3.7% of cases). 91.4% were estrogen receptor (ER) positive and 82.7% were progesterone receptor (PR) positive. Triple negative receptor status was found in 8.9% cases. Bilateral breast cancer was diagnosed in two patients (1.2%) (Table 1).

Multiple primary cancers were diagnosed in 20 of 165 patients (12.1%). Prostate cancer was diagnosed in five cases (3.0%) and colorectal cancer was diagnosed in four patients (2.4%). Approximately one-half of the patients had a positive family history of cancer: 31.5% for breast and/or ovarian cancer and 36.4% for other cancers. The clinical features of breast cancer diagnosed in carriers of mutations and non-carriers are presented in Table 1.

We did not find statistically significant differences between mutation carriers and non-carriers (*p* > 0.5), except for a higher incidence of bilateral breast cancer in *PALB2* mutation carriers (*p* = 0.004).

One of twenty founder mutations was diagnosed in 22 of 165 men (13.3%) (Table 2). A *CHEK2* mutation was found in 14 patients (8.5%). The most commonly detected *CHEK2* allele was the missense c.470C > T mutation, which was found in nine subjects (5.5%). A protein truncating mutation in *CHEK2* gene was found in five patients (3.0%). A *PALB2* mutation was seen in four patients (2.4%). A *BRCA2* mutation was found in two patients (1.2%) and a *BRCA1* mutation was found in only one patient (0.6%). One mutation was found in *NBN* (0.6%) and no mutations were found in *RECQL.*

**Table 2:** The frequencies of mutation detected in study in a series of 165 male breast cancer patients by family history of breast and ovarian cancer

Mutations	All patients (***n*** = 165)		Family History Positive (***n*** = 52)		Family History Negative (***n*** = 113)		Chi-square test
	N	%	N	%	N	%	p
**Any*BRCA1***	**1**	**0.6**	**1**	**1.9**	**0**	**0.0**	**0.14**
c.5263_5264insC	1	0.6	1	1.9	0	0.0	
c.181 T > G	0	0.0	0	0.0	0	0.0	
c.4035delA	0	0.0	0	0.0	0	0.0	
c.3700_3704delGTAAA	0	0.0	0	0.0	0	0.0	
c.68_69delAG	0	0.0	0	0.0	0	0.0	
c.5251C > T	0	0.0	0	0.0	0	0.0	
c.3756_3759delGTCT	0	0.0	0	0.0	0	0.0	
**Any*BRCA2***	**2**	**1.2**	**1**	**1.9**	**1**	**0.9**	**0.57**
c.658_659delGT	1	0.6	1	1.9	0	0.0	
c.5946delT	1	0.6	0	0.0	1	0.9	
c.3847_3848delGT	0	0.0	0	0.0	0	0.0	
c.5239_5240insT	0	0.0	0	0.0	0	0.0	
c.7913_7917del5	0	0.0	0	0.0	0	0.0	
**Any*PALB2***	**4**	**2.4**	**3**	**5.8**	**1**	**0.9**	**0.57**
c.509_510delGA	4	2.4	3	5.8	1	0.9	
c.172_175deITTGT	0	0.0	0	0.0	0	0.0	
**Any*CHEK2***	**14**	**8.5**	**4**	**7.7**	**10**	**8.8**	**0.80**
***CHEK2*truncating**	**5**	**3.0**	**2**	**3.85**	**3**	**2.7**	**0.68**
c.100delC	3	1.8	1	1.9	2	1.8	
c.444 + 1G > A	1	0.6	1	1.9	0	0.0	
del5395(ex10-11del)	1	0.6	0	0.0	1	0.9	
c.470C > T	9	5.5	2	3.85	7	6.2	
***NBN*** c.657_661delACAAA	**1**	**0.6**	**0**	**0.0**	**1**	**0.9**	**0.50**
***RECQL*** c.1667_1667 + 3delAGTA	0	0.0	0	0.0	0	0.0	–

*BC* breast cancer, *OV* ovarian cancer

A mutation was found in 20% of men diagnosed before age 50 and in 12.9% of men diagnosed after age 50. A mutation was found in 9.7% of men with ductal carcinoma and 15.2% of men with other histopathological subtypes. Mutations were more common in familial cases (17.3%) than in non-familial cases (11.5%).

Compared to the frequency of mutations in cancer-free controls from our previous studies, we observed statistically significant associations for *PALB2* (OR = 11.66 *p* < 0.001) and for *CHEK2* truncating mutations (OR = 2.93; *p* = 0.02) (Table 3).

**Table 3:** Association of selected alleles in *BRCA1, PALB2, CHEK2* and *NBN* with risk of male breast cancer

Gene	Mutation	All patients			Control^**a**^					
		Positive	Total	%	Positive	Total	%	OR	95%CI	***p***-value
***BRCA1***	c.5263_5264insC	1	165	0.6%	13	4000	0.32%	1.87	0.24–14.38	0.54
	c.181 T > G	0	165	0.0%	3	4000	0.08%	–	–	
	c.4035delA	0	165	0.0%	1	4000	0.03%	–	–	
	Any BRCA1	1	165	0.6%	17	4000	0.42%	1.43	0.19–10.80	0.73
***PALB2***	**509_510delGA**	**4**	**165**	**2.4%**	**7**	**4702**	**0.1%**	**16.70**	**4.83–57.50**	**< 0.001**
	172_175deITTGT	0	165	0.0%	3	4702	0.05%	–	–	
	**Any PALB2**	**4**	**165**	**2.4%**	**10**	**4702**	**0.15%**	**11.66**	**3.62–37.57**	**< 0.001**
***CHEK2***	**1100delC**	**3**	**165**	**1.8%**	**12**	**5496**	**0.2%**	**8.46**	**2.37–30.29**	**0.001**
	c.444 + 1G > A	1	165	0.6%	22	5496	0.4%	1.52	0.20–11.32	0.68
	del5395 (ex10-11del)	1	165	0.6%	24	5496	0.4%	1.39	0.19–10.34	0.75
	**CHEK2 truncating**	**5**	**165**	**3.0%**	**58**	**5496**	**1.1%**	**2.93**	**1.16–7.40**	**0.02**
	c.470C > T	9	165	5.5%	264	5496	4.8%	1.28	0.59–2.17	0.84
	Any CHEK2	14	165	8.5%	321	5496	5.8%	1.45	0.87–2.42	0.21
***NBN***	c.657_661delACAAA	1	165	0.6%	22	4000	0.55%	1.10	0.15–8.23	0.92

In two men, three primary cancers were diagnosed (1.3%). Both of these patients were diagnosed with bilateral breast cancer and both carried a mutation in the *PALB2* gene.

^a^- for BRCA1, CHEK2, PALB2, NBN mutation frequencies in cancer-free controls were from our previous studies [29, 30, 56].

## Discussion

A founder mutation of *BRCA1*, *BRCA2*, *CHEK2*, *PALB2* and *NBN* was found in 13.3% of men with breast cancer in Poland. The mutation frequency was similar in familial and non-familial cases and in young men and older men.

This study supports the recommendation that a panel test be offered to all Polish men with breast cancer. In a previous study we have shown that 20 founder mutations account for 85% of all mutations in Polish women with hereditary breast cancer and were detected in 46% cases [27].

We identified a strong association with *PALB2* mutation and male breast cancer (OR = 11.6). In previous reports from other ethnic groups, the frequency of *PALB2* mutations ranged from 0.8 to 6.4% [12, 19, 21, 23–26].. Two previous studies reported an association of *PALB2* mutations and breast cancer risk in men [21, 23]. Pritzlaff et al. reported a 6.6-fold increased risk (*p* = 0.013) in an international cohort of 715 male breast cancer patients [21].

Our study is the first that evaluated the frequency of mutations in the *PALB2* gene in men with breast cancer from Poland, which is populated by ethnic Slavs. In the current study two patients with a *PALB2* mutation were diagnosed with bilateral breast cancer and they were diagnosed with the third primary cancer. One of these had esophageal cancer and the other had bladder cancer. In previous studies, three male breast cancer patients with a *PALB2* mutation had a second primary cancer (thyroid cancer, melanoma, prostate) [16, 21]. Male breast cancer patients who carry a mutation in P*ALB2* gene may be candidates for screening for second primary malignancies.

We have previously reported relatively poor survival for women with breast cancer and a *PALB2* mutation [30]. Female breast cancer patients who carried one of the two Polish *PALB2* founder mutations had a 10-year survival of 48%, compared to 75% for patients with breast cancer without a PALB2 mutation (HR = 2.27, 95% CI 1.64–3.15; *p* < 0.0001). Studies from Finland and China also reported poor survival of *PALB2*-associated breast cancer patients [57, 58]. In our cohort, 3 of 4 men with breast cancer and a *PALB2* mutation died of metastatic cancer. The first patient was diagnosed with bilateral breast cancer at age 52 and died at age 72 of esophageal cancer. The second was diagnosed with unilateral breast cancer at age 42 and died of metastatic breast cancer at age 53. The third patient had bladder cancer and bilateral breast cancer at age 77 and died of breast cancer at age 79. The fourth patient was diagnosed with breast cancer at age of 42 (in 2017) and is currently alive. This data suggests poor prognosis in men with breast cancer and a *PALB2* mutation but larger studies are necessary.

We observed a correlation between the three truncating mutation in the *CHEK2* gene (c.1100delC, c.444 + 1G > A and del5395(ex10-11del)) and male breast cancer in Poland (OR = 2.93: *p* = 0.02). The 1100delC mutation in *CHEK2* has been evaluated in several other studies [59–63] and four of these showed an increased risk of male breast cancer. In Finland, Hallamies at al. reported an odds ratio of 4.5 for men with the 1100delC allele (p = 0.02) [22]. A study in Netherlands confirmed the increased risk (OR: 4.1, *p* = 0.005) [20]. In the largest study of 715 male breast cancers the odds ratio associated with the 1100delC mutation in the *CHEK2* gene was 3.8 (*p* = 0.002) [21]. The frequency of the c.1100delC allele in *CHEK2* gene varies widely in different populations; the frequency is high in Finland (1.1–1.4%) and the Netherlands (1.3–1.6%) but is lower in Poland (0.2%). As a consequence the contribution of this allele to the burden of male breast cancer varies widely from country to country [20, 22, 56].

We found that the frequency of the 1100delC founder mutation in the *CHEK2* gene to be higher in male breast cancer cases (1.8%) than in females with breast cancer (0.6%) [29]. A similar result was obtained by Hallamies at al. in Finland. The observed frequency of this mutation was higher in male breast cancer patients (5.9%) then among female patients (2.0%) [22, 64].

We did not find a significant association between the c.470C > T mutation in the *CHEK2* gene and the risk of male breast cancer (*p* = 0.84). However, multiple primary cancers were diagnosed in three of nine carriers of this mutation (33.3%)(rectal cancer, myeloid leukemia and skin squamous cell carcinoma) In a previous study we reported a correlation between the c.470C > T mutation in the *CHEK2* gene and multi-organ cancer susceptibility (breast, colon, kidney, prostate and thyroid cancer) with odds ratios ranging between 1.5 and 2.0 [65].

*PALB2* and *CHEK2* are clear breast cancer susceptibility genes. *PALB2* encodes a BRCA2-binding protein that acts as a linker between BRCA1 and BRCA2 to form a BRCA1-associated genome surveillance complex. The complex is essential for the homologous DNA break repair [66]. Homozygous mutations of *PALB2* cause Fanconi anemia, a rare recessive chromosomal breakage syndrome characterized by physical abnormalities, bone marrow failure and a high risk of malignancy. Heterozygous carriers of *PALB2* mutations are at increased risk of breast and pancreatic cancers [67, 68].

CHEK2 is involved in the p53 pathway of DNA damage responses. CHEK2 interacts with many different proteins. Upon ionizing radiation-induced DNA damage, CHEK2 is activated by ataxia telangiectasia mutated (ATM) and is in turn capable of phosphorylating several substrates including Cdc25A, Cdc25C, p53, and BRCA1, leading to cell cycle arrest, apoptosis and DNA repair [69].

In contrast to our findings in female breast cancer patients, there were relatively few mutations found in *BRCA1* and *BRCA2.* Several previous studies reported an association of *BRCA1* and *BRCA2* mutations with breast cancer risk in men [5–18]. In these studies, the frequency of detected mutations in the *BRCA2* gene was higher than seen in the *BRCA1* gene [14–16, 18, 21].

There are several limitations to this study. Patients were selected from a registry of breast cancer cases held at the Hereditary Cancer Center in Szczecin which could have over-represented patients with a positive history of breast and ovarian cancer. The molecular analysis was based on recurrent mutations which may underestimate the total mutation frequency.

## Conclusions

Our study shows that mutations in the *CHEK2* and *PALB2* genes are important risk factors for male breast cancer in Poland. We recommend that a simple test for Polish founder mutations in *BRCA1, BRCA2, PALB2* and *CHEK2* should be offered to all male breast cancer patients in Poland.

## Supplementary Information

**Additional file 1.**

## Data Availability

All data generated or analysed during this study are included in this published article [and its supplementary information files].

## Abbreviations

ASA-PCR: allele-specific amplification PCR
ATM: ataxia telangiectasia
BRCA1: breast cancer 1 gene
BRCA2: breast cancer 2 gene
Cdc25: M-phase inducer phosphatase 1
CHEK1: checkpoint kinase 1 gene
CHEK2: checkpoint kinase 2 gene
CI: confidence interval
ER: estrogen receptor
HER2: human epidermal growth factor receptor 2
NBN: Nijmegen breakage syndrome gene
OR: odds ratio
PALB2: partner and localizer of BRCA2 gene
PCR: polymerase chain reaction
PR: progesteron receptor
RECQL: RecQ protein-like gene
RFLP-PCR: restriction fragment length polymorphism PCR
